# Insight into Nrf2: a bibliometric and visual analysis from 2000 to 2022

**DOI:** 10.3389/fgene.2023.1266680

**Published:** 2023-09-15

**Authors:** Yawei Ma, Zhongqing Wang, Yuedong Hu

**Affiliations:** ^1^ Department of Ophthalmology, The First Hospital of China Medical University, Shenyang, China; ^2^ Department of Information Center, The First Hospital of China Medical University, Shenyang, China

**Keywords:** Nrf2, bibliometric analysis, VOSviewer, citespace, visualization

## Abstract

**Background:** Nrf2 plays a pivotal role in governing the antioxidant defense system, triggering the transcription of diverse genes involved in cellular protection. Its role in mitigating oxidative damage and modulating inflammatory processes has made Nrf2 an attractive target for therapeutic interventions. Despite the growing interest in Nrf2 research, a bibliometric analysis is relatively rare. This study aimed to clarify Nrf2’s role in multiple diseases, identify emerging trends and hotspots using bibliometric analysis, and provide valuable insights and potential directions for future therapeutic interventions.

**Methods:** The Science Citation Index of Web of Science Core library from 2000 to 2022 was searched on 22 October 2022. Use Microsoft Excel, CiteSpace, Bibliometrix, and VOS viewers for data collection and visualization of research focus and trends.

**Results:** A vast collection of 22,040 research studies on Nrf2 published between 2000 and 2022 were identified. Nrf2 research has seen significant growth globally from 2000 to 2022. China leaded in publication numbers (9,623, 43.66%), while the United States dominated in citation frequency with 261,776 citations. China Medical University was the most productive institutions (459, 2.08%). Masayuki Yamamoto topped in publications (307), while Itoh K. ranked first in citations with 3669. Free Radical Biology and Medicine was the journal with the most studies and citations on Nrf2 (613, 29,687 citations). The analysis of keyword clustering enhanced the categorization of topics and can be summarized as oxidative stress, cancer, disorders in glycolipid metabolism, inflammation, and neurological conditions.

**Conclusion:** China and the United States are the pioneers in Nrf2 research. Recently, there has been a comprehensive exploration of Nrf2 involving both experimental and clinical aspects, as well as mechanisms and therapeutic applications. Investigating novel molecular mechanisms, including NF-κB, Ho1, and Keap1, and developing enhanced, targeted Nrf2 activators or inhibitors to uncover the interplay among cancer, glycolipid metabolic disorder, inflammation, and neurological disorders will be upcoming trends and hotspots.

## 1 Introduction

Nrf2, or nuclear factor erythroid 2-related factor 2, is a crucial transcriptional factor involved in antioxidant response and has significant implications in inflammation and cellular defense against oxidative stress. Nrf2 is primarily located in the cytoplasm under basal conditions, where it forms a complex with its inhibitor, Keap1. However, under oxidative stress, the degradation of Nrf2 decreased rapidly, resulting in the accumulation and nuclear translocation of Nrf2, where it binds to antioxidant response elements (AREs) in the promoter region of a group of antioxidant and cellular defense targets. The binding can transcriptionally activate various enzymes and proteins, thereby regulating metabolic and inflammatory responses in the body (Kobayashi et al., 2009; Dodson et al., 2019). Nrf2 has been studied in numerous diseases, gaining a comprehensive understanding of Nrf2 and its regulatory mechanisms is beneficial for developing novel therapeutic approaches to address diseases associated with oxidative stress.

Bibliometric analysis is a method that utilizes software to visualize the distribution, connections, and trends of countries, institutions, authors, journals, and research areas. It provides valuable predictions for the future development of a specific field by offering insights into hotspots and trends (Ma et al., 2022). This approach has gained popularity as a research framework for assessing impact and evidence, supported by the growing accessibility of research data through databases like Web of Science (WOS) (Chen et al., 2020). While basically no bibliometric studies have been conducted on Nrf2, this paper aims to utilize bibliometric analysis to examine the literature on Nrf2 over the past two decades. This study aims to comprehend Nrf2’s characteristics, forecast research trends and hotspots, explore international collaboration networks, and provide vital information to advance future investigations in the Nrf2 field, benefiting clinicians and researchers.

## 2 Materials and techniques

### 2.1 Data collection and search scheme

**FIGURE 1:**
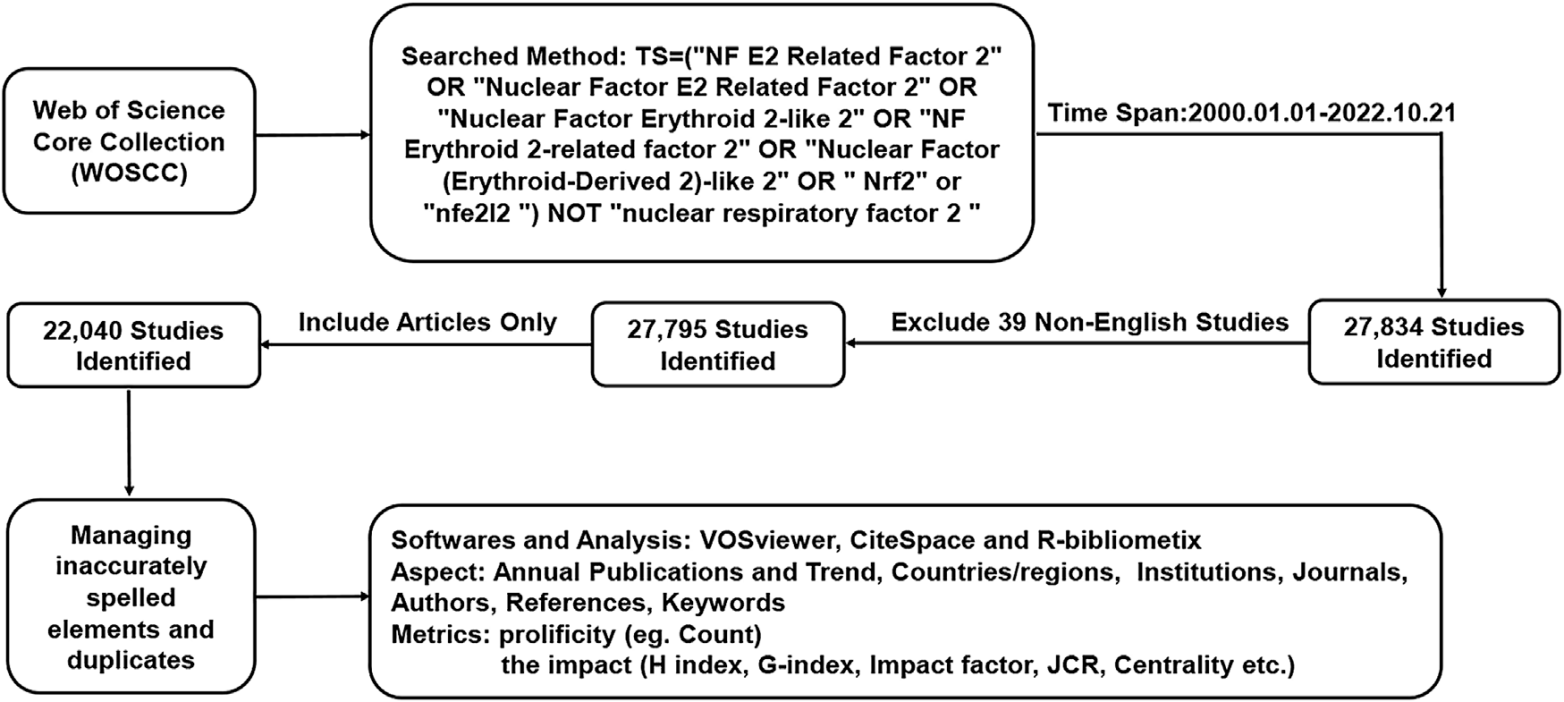
A diagram depicted the sequential evaluation and selection steps. The study encompassed English-language articles published between 2000 and 2022, with the removal of any duplicate publications.

We chose to use the database of the Web of Science (WOS) core collection provided by Clarivate Analytics for bibliometric analysis because it is widely considered to be the most suitable database for this type of analysis (Ke et al., 2020). To prevent daily updating bias, we conducted the analysis within a single day, taking advantage of the fact that the database remains accessible at all times. Our method for conducting the search in the WOSCC database was as outlined below: TS = (“NF E2 Related Factor 2”OR “Nuclear Factor E2 Related Factor 2”OR “Nuclear Factor Erythroid 2-like 2”OR “NF Erythroid 2-related factor 2”OR “Nuclear Factor (Erythroid-Derived 2)-like 2”OR “Nrf2” OR “nfe2l2”) NOT (“nuclear respiratory factor 2”). The study specifically targeted publications published between 1 January 2000, and 21 October 2022. We excluded any literature not written in English and only included articles for analysis, resulting in 22,040 articles that met our criteria (as illustrated in Figure 1). These publications were downloaded in TXT format on 22 October 2022, for further analysis.

### 2.2 Data gathering and refining

The initial dataset obtained through the designated methodology consisted of key metrics such as the number of papers, citation count, H-index, and relevant data related to affiliations, authors, journals, and geographical information. Following that, we excluded any inaccurately spelled items and eliminated duplicate authors from our analysis. It is worth mentioning that cited references can exist in multiple versions or variations and it is possible for multiple authors to share identical name abbreviations, while cited journals may have distinct formats, all of which may result in inaccuracies in the analysis. So we organized the data manually to prevent any duplication. We successfully managed the issue of inaccurately spelled elements and duplicates by utilizing a thesaurus file. For example, we substitute “Taiwan”/“Hong Kong”/“People R China” with the term “China”. This resource enabled us to consolidate repeated entries, eliminate redundant words, and rectify any spelling errors in a methodical approach. Subsequently, we utilized VOSviewer (version 1.6.18.0), CiteSpace (version 6.1. R6), and the “bibliometrix package 4.1.2”of R software (version 4.2.3) to analyze the refined dataset from a bibliometric perspective.

### 2.3 Bibliometric analysis and visualization

The publication quantities and their respective citations are obtained through the built-in capability in WOSCC. Among them, the H-index functions as a quantitative measure to evaluate the scholarly achievements or research productivity of individuals (Hirsch, 2005). Originally, the H-index was introduced to assess the scholarly contributions of individual researchers. Over time, its scope has broadened to include assessing research groups, institutions, and research communities on a national or regional scale, as well as its application in evaluating academic journals. On the other hand, the G-index measures the maximum number of publications that have achieved a citation count equal to or higher than the H-index (Abbas, 2012). Furthermore, we use the impact factor (IF) and the latest edition of Journal Citation Reports (JCR) as important indicators to evaluate the scientific value of research (Eyre-Walker and Stoletzki, 2013).

Various software tools were employed for conducting bibliometric analysis in the study. To visualize and construct bibliometric networks, we relied on the VOSviewer software, developed by Leiden University in the Netherlands. This software facilitated in-depth analysis of bibliographic coupling, co-citation, co-occurrence and international collaborations (van Eck and Waltman, 2017). Due to its capacity to map and detect clusters in a network simultaneously, the software was used in combination with clustering methods to divide networks into separate clusters, taking into account the intensity of connections between nodes. Additionally, we applied the R4.1.2-based “bibliometrix” to generate visualizations describing the relationships between different journals and conduct three-field plot analysis. A “three field plot” is a graphical representation that visually depicts the relationships among three distinct entities, it provides insights into the global nature of research collaborations and the distribution of research activities in a given field. Overall, we use “three field plot” as an effective tool for visually summarizing complex relationships and interactions among countries, institutions, and authors in research related to Nrf2. Furthermore, CiteSpace, a software tool created by Professor (Chen et al., 2009), was employed in the analysis to produce dual-map overlays for journals, perform cited keywords analysis, and identify references exhibiting notable citation bursts. The dual-map function helped depict the subject distribution of academic journals by placing citing journals on one side and cited journals on the other, illustrating their relationship. The size of nodes in the network was determined by the parameters weight, which includes the number of publications, number of citations, or frequency of occurrence. Larger nodes were attributed to higher weight. Each node and line in the network were assigned a color representing its respective cluster. The lines between nodes represented the connections among them. Moreover, the Total Link Strength (TLS) was utilized to evaluate the total co-authorship and co-citation links among different countries, institutions, and authors.

VOSviewer and CiteSpace are both tools used for bibliometric analysis. They share the common purpose of visually revealing literature networks, collaborative relationships, and thematic trends. VOSviewer emphasizes co-occurrence analysis and customizable network visualization to understand keyword associations and collaborations. CiteSpace focuses on temporal analysis and citation networks to uncover research history and impact. Employing both tools in this study may offer a more thorough comprehension. In addition to that, online tools facilitate the generation of chord diagrams of countries. These diagrams involve dividing the circle based on proportional country distribution and linking interrelated nations through chord segments to portray their connections. The width of these segments can convey the intensity of these associations.

## 3 Results

### 3.1 Annual publications and trend

**FIGURE 2:**
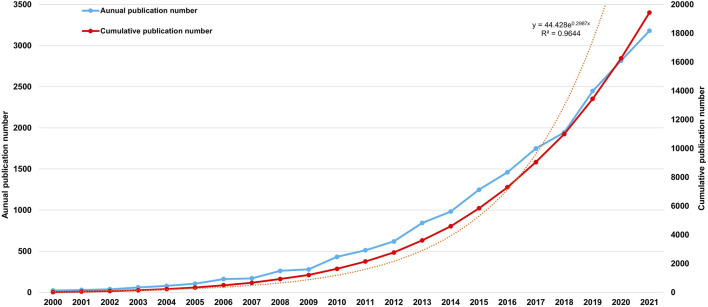
Nrf2-related Publication volume overview. The annual publication number and cumulative publication number during 2000 and 2021.

According to our search strategy, overall 22,040 literature pieces were gathered spanning from 2000 to 2022. The yearly publication outcomes related to Nrf2 are presented in Figure 2, excluding incomplete data for articles published in 2022. The interest in Nrf2 has been increasing significantly across the most recent 20 years. In 2000, there were only 22 publications related to Nrf2, which increased to 1,248 in 2015, and reached a record high of 3,181 in 2021. Since 2018, there has been a significant expansion in the amount of published articles. The overall global publications in 2021 on this topic are almost 144.59 times greater than those in 2000, which is a remarkable achievement. The fast-paced growth of publication number indicates a rising interest among scholars in this field, which means scholars conducting research and the flourishing of theories related to Nrf2 are evident.

### 3.2 Contribution of countries/regions

Research on Nrf2 has gained global attention, with ongoing studies conducted in 117 countries and regions worldwide. The publication count was used to determine the ranking of the top 10 countries/regions, and it also includes information on their citation frequency, H-index and centrality. The centrality of each country/region indicates its importance in the network (as seen in Table 1). Centrality measures the intensity of links among nodes within a network, serving as an indicator of the importance and impact. The top 10 countries/regions that have published over 500 papers on Nrf2 are mainly from Asia, Europe and North America. China leads the pack with 9,594 counts (43.53%) in terms of publication numbers, followed by the United States with 4,931 counts (19.92%) and South Korea with 1,844 counts (8.37%). The combined publications from China and the United States make up 66.03% of the total, which is significantly higher than any other country.

**TABLE 1:** The top 10 countries with the most publications on Nrf2.

Rank	Countries/regions	Counts	Citing articles	Citations	Average citations	H-index	Centrality
1	People R China	9623	102061	179971	19	118	0.03
2	United States	4931	129240	261776	53	208	0.16
3	South Korea	1844	35969	48747	26	91	0.13
4	Japan	1540	43956	88410	57	142	0.31
5	Germany	781	28024	33944	43	88	0.64
6	Italy	726	18573	21657	30	68	0.24
7	India	708	14591	16859	24	62	0.00
8	Taiwan	622	13060	15524	25	60	0.03
9	Egypt	618	6400	8155	13	42	0.00
10	England	597	23749	30979	52	86	0.45

The United States is at the forefront in terms of citation frequency, with a remarkable total of 261,776 citations, followed by China (179,971) and Japan (88,410). Significantly, the United States had a citation count of 53 per publication, highlighting its strong impact. In contrast, China had a high citation count but a relatively low citation/publication ratio compared to the top 10 countries, standing at 19. Notably, England demonstrated a high citation/publication ratio of 52, indicating the high quality of its published papers despite a relatively lower publication count. With regards to the H-index, the United States held the top position with a score of 208, while Japan and China achieved H-indices of 142 and 118, respectively.

**FIGURE 3:**
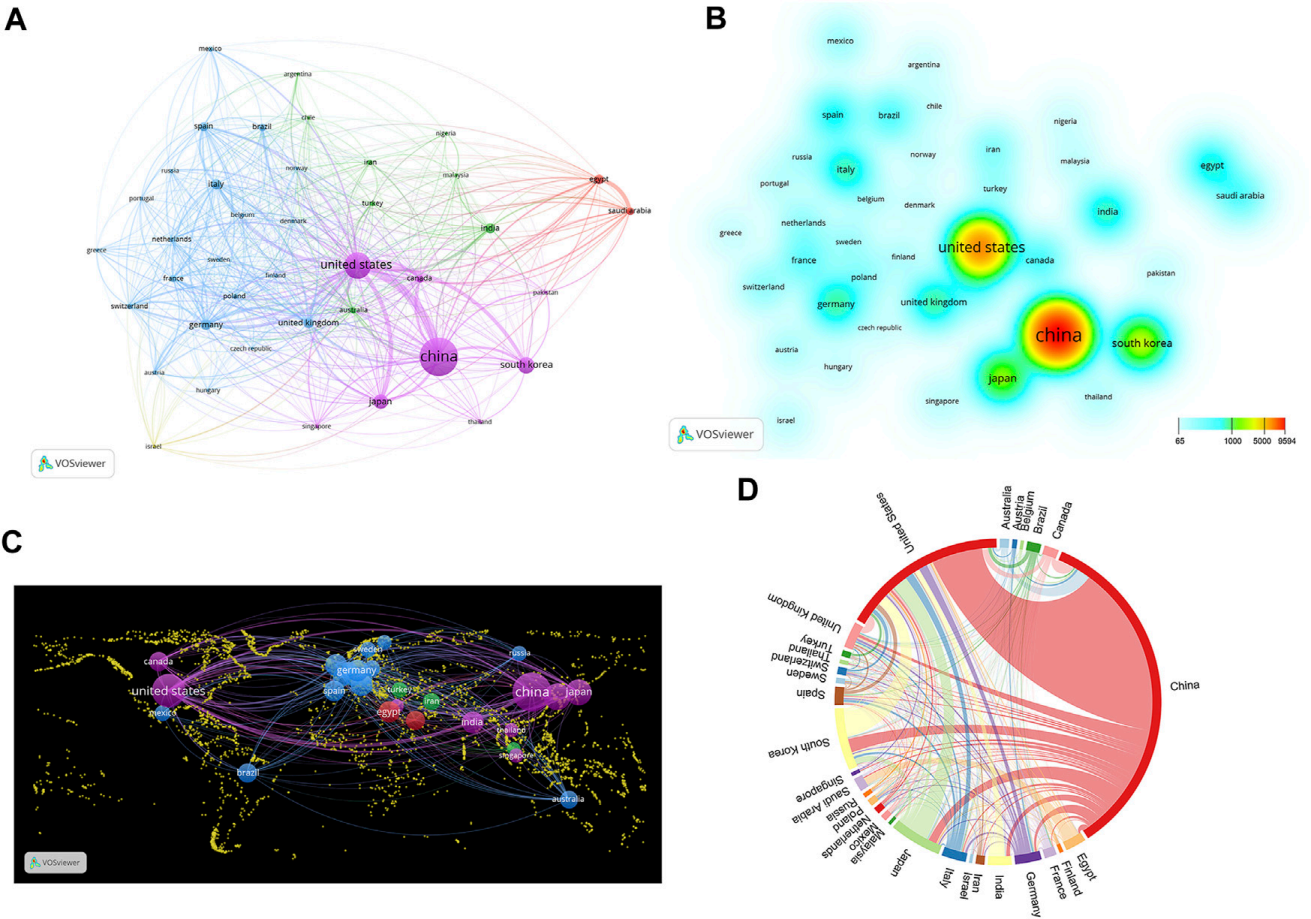
The involvement of various countries in Nrf2 research. **(A)** Nrf2 research collaboration map across countries. Countries are represented by circles, while lines illustrate their collaborations. The weight corresponds to the publication count, line thickness denotes the strength of collaboration, and distinct colors highlight clusters. **(B)** Density map of countries. The evolution of colors demonstrates the volume of national publications. **(C)** World map illustrating the density of cooperation among countries. **(D)** A chord diagram assessing the global collaboration among clusters.

Figures 3A, B provide insights into the collaborative efforts among the top 39 countries based on their publication output. The analysis highlights that these countries can be classified into five distinct clusters in VOSviewer, reflecting varying degrees of collaboration. These clusters are visually represented using different colors. Figure 3C illustrates the collaborative relationships among the countries mentioned earlier in the form of a world map. The top 30 countries are represented using a chord diagram, where each country is assigned a distinct color. The entire circle is divided proportionally based on each country’s representation, the wider the chord segments, the stronger the collaborative relationships among these countries are (shown in Figure 3D). In conclusion, the majority of collaboration was centered around China and the United States, followed by Japan, South Korea, Germany and the United Kingdom, were the primary focus of collaboration, whereas there was a limited level of collaboration with other countries. And the metrics used in this study are listed in Table 2.

**TABLE 2:** Metrics used in the research.

Metrics	Information or equation	Application
Centrality	https://www.webofscience.com	Indicators reflecting the importance and influence in the academic field
Average citation number	Average citation number = $\frac{\text{total}\;\text{citation}\;\text{count}}{\text{total}\;\text{number}\;\text{of}\;\text{documents}}$	Impact and popularity of the literature
H index	N papers have each been cited at least N times	Evaluations in academia span various levels: individuals, research teams, institutions, research communities, and academic journals
G-index	The average number of citations for G papers is at least G	An important indicator of academic impact
Impact factor	IF_y_ = $\frac{{\text{Citations}}_{y-1}+{\text{Citations}}_{y-2}}{{\text{Publications}}_{y-1}+{\text{Publications}}_{y-2}}$	An essential measure of a journal’s influence
JCR	https://jcr.clarivate.com/jcr	An essential indicator of a journal’s quality

### 3.3 Contributions of institutions

The analysis of research institutions can be used to determine the global distribution of Nrf2 research and to identify potential collaboration partners. In Table 3, you can find the top 10 institutions, with China Medical University being the most productive (459, 2.08%), followed by the Chinese Academy of Science (343, 1.56%) and Tohoku University (324, 1.47%). China dominated the top 10 institutions, with the majority (8 out of 10) being from this country, representing 11.26% of the total papers. Johns Hopkins University stands out with the highest average citations (107.7933) and H-Index (108), followed closely by Tohoku University with an average citation of 82.5957 and an H-Index of 97. However, the other institutions have average citations below 30 and H-Index below 60.

**TABLE 3:** The top 10 institutions conducting Nrf2 by volume.

Rank	Institution	Country	Counts	Citations	Average citations	H-index	Centrality
1	China Medical University	China	459	10723	23.3617	43	0.05
2	Chinese Academy of Science	China	343	10252	29.8892	57	0.26
3	Tohoku University	Japan	324	26761	82.5957	97	0.09
4	Nanjing Medical University	China	303	5020	16.5677	38	0.10
5	Johns Hopkins University	United States	300	32338	107.7933	108	0.06
6	Jilin University	China	290	8132	28.0414	52	0.07
7	Zhejiang University	China	290	6491	22.3828	43	0.03
8	Shandong University	China	271	6189	22.8376	42	0.01
9	Shanghai Jiao Tong University	China	264	5608	21.2424	40	0.03
10	Sun Yat-sen University	China	262	7003	26.729	43	0.02

**FIGURE 4:**
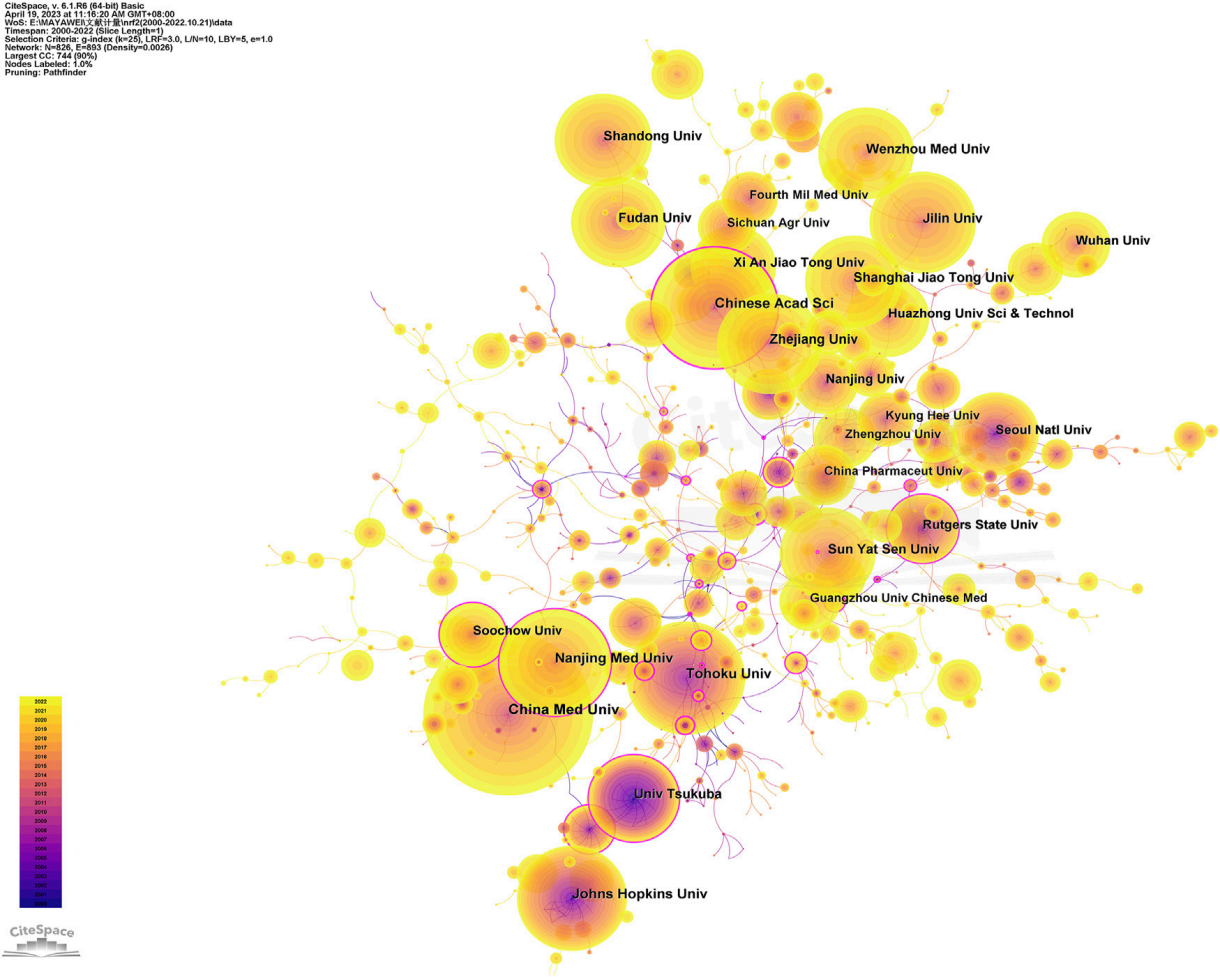
Visualization of the institutions involved in research on Nrf2. The study encompassed English-language articles published between 2000 and 2022, with the removal of any duplicate publications.

Figure 4 generated using CiteSpace, depicts an institutional co-occurrence map illustrating the leading 10 institutions in Nrf2 research. The map emphasizes their publication contributions and prominence within the field. The visualization represents institutions as nodes, with their sizes reflecting their significance in the field. The connections between nodes indicate collaboration, and the links are color-coded to represent the specified time period. If a node has a centrality exceeding 0.1, it indicates that the node is a central node, which is relatively important and has a significant influence in the study. These nodes are marked with purple circles.

### 3.4 Journal of high yield

**FIGURE 5:**
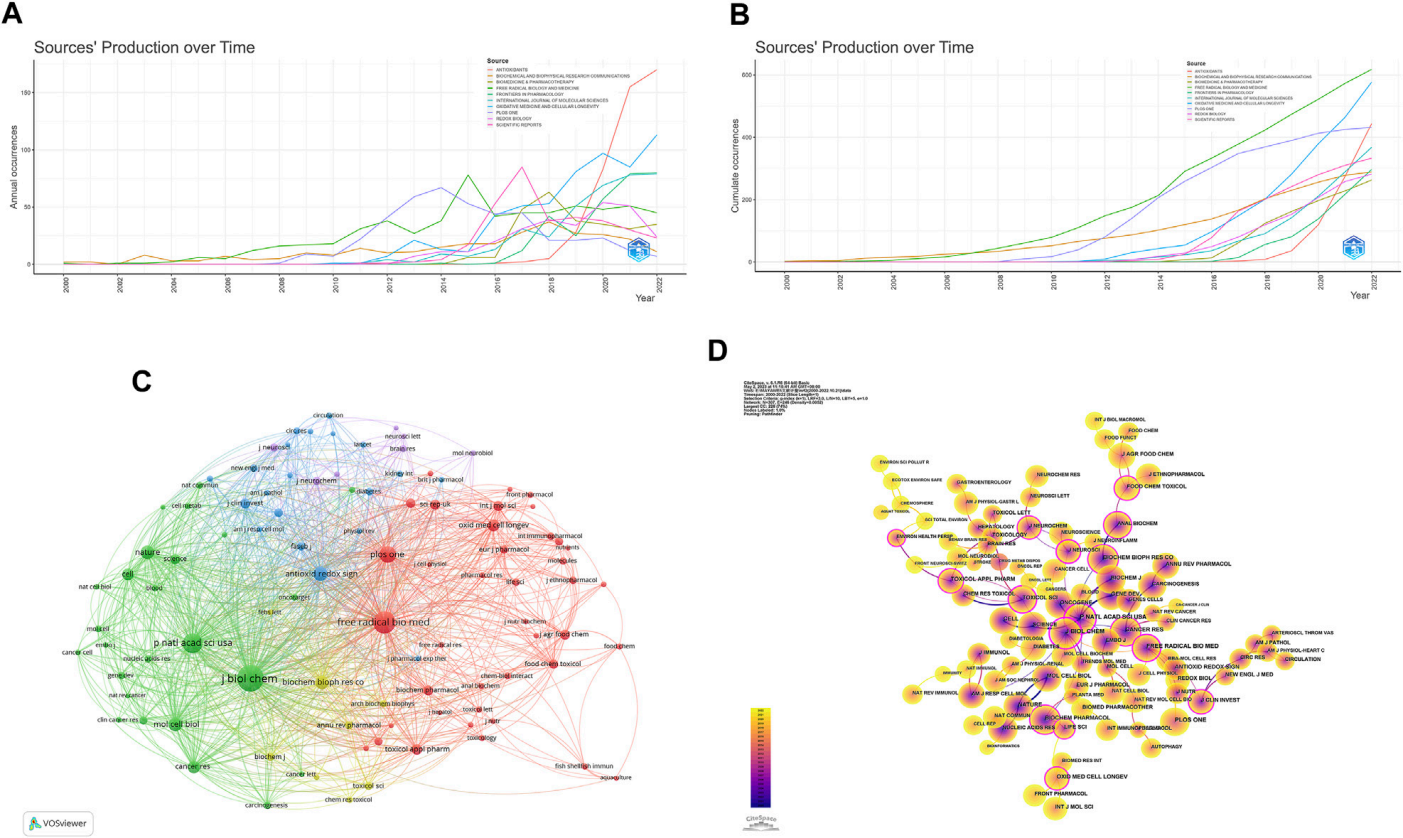
Publications and co-citation network of journals to the field of Nrf2 research. **(A)** Annual publications of the top 10 journals. **(B)** Cumulative publications of the top 10 journal. **(C)** The network of collaboration among the top 100 co-cited journals. The nodes indicate the number of citations, while the links represent the intensity of cooperation. **(D)** The journals co-citation network clustering. The use of different colors for nodes and links in the visualization represents the chronological occurrence of co-citation relationships.

There has been a total of 1,927 academic journals that have published articles related to Nrf2. The leading 20 journals in terms of publishing articles on Nrf2 are presented in Table 4, making up 27.00% of all the articles. The annual and cumulative publications of the journal are displayed in the Figures 5A, B. The publication volume of Antioxidants has experienced a significant increase in the past 2 years, showing a steep upward trend. Free Radical Biology and Medicine holds the top spot for the highest number of published studies on Nrf2, (8.101, Q1) (613). This is followed by Oxidative Medicine and Cellular Longevity (7.310, Q2) (576), and Antioxidants (7.675, Q1) (441). Among the top 20 journals based on publication count, half of them is classified as Q1 in the Journal Citation Reports (JCR), while 70% of these journals have an Impact Factor (IF) exceeding 5. Remarkably, The Journal of Biological Chemistry demonstrated the highest H-index (92) and G-index (165), closely followed by Free Radical Biology and Medicine with an H-index of 89 and a G-index of 132. Plos One ranked third with an H-index of 69 and a G-index of 99, suggesting the inadequate quality of a considerable number of studies.

**TABLE 4:** The top 20 journals by publication volume in the domain of Nrf2 research.

Rank	Journal	Counts	Citations	Average citations	H-index	G-index	JCR	If (2021)
1	Free Radical Biology and Medicine	613	29687	48.43	89	132	Q1	8.101
2	Oxidative Medicine and Cellular Longevity	576	9695	16.83	47	62	Q2	7.310
3	Antioxidants	441	2576	5.84	23	29	Q1	7.675
4	Plos One	432	16990	39.33	69	99	Q2	3.752
5	International Journal of Molecular Sciences	361	4419	12.24	34	44	Q1	6.208
6	Scientific Reports	334	8596	25.74	48	68	Q2	4.997
7	Frontiers in Pharmacology	294	3315	11.28	30	39	Q1	5.988
8	Biochemical and Biophysical Research Communications	288	8160	28.33	44	73	Q3	3.322
9	Redox Biology	278	8407	30.24	47	73	Q1	10.787
10	Biomedicine and Pharmacotherapy	263	5437	20.67	39	51	Q1	7.419
11	The Journal of Biological Chemistry	244	29379	120.41	92	165	Q2	5.485
12	Toxicology and Applied Pharmacology	240	9994	41.64	57	84	Q2	4.460
13	International Immunopharmacology	237	5085	21.46	40	51	Q1	5.714
14	Food and Function	233	3611	15.5	33	45	Q1	6.317
15	Food and Chemical Toxicology	219	7094	32.39	47	72	Q1	5.572
16	Life Sciences	189	3283	17.37	29	45	Q1	6.780
17	European Journal of Pharmacology	184	4467	24.28	38	56	Q2	5.195
18	Molecules	183	2381	13.01	29	38	Q2	4.927
19	Journal of Functional Foods	175	2186	12.49	23	35	Q2	5.223
20	Molecular Medicine Reports	167	2369	14.19	38	56	Q3	3.423

Figure 5C displays the Top 100 co-citation journal network, consisting of 5 clusters, which was created using VOSviewer. The red cluster, which is the largest among the clusters, consists of 40 journals that primarily specialize in Biochemistry, Pharmacology, Toxicology, and Food Science, with a particular emphasis on Immunology and Molecular Sciences. Notably, Free Radical Biology and Medicine holds a prominent position within this cluster. This journal has published a considerable amount of basic and clinical research, focusing primarily on the mechanisms underlying altered metabolism and redox signaling.

The journals represented by the green cluster cover research areas such as cancer, autophagy, cell metabolism, gene and development, and nucleic acids. Among them, the Journal of Biochemistry occupies the central position in the co-citation network with the highest quotation number and strong co-citation intensity. This journal primarily focuses on molecular and cellular processes and fundamental mechanisms.

Antioxidants and Redox Signaling and Journal of Clinical Investigation hold a prominent position within the blue cluster. This cluster primarily encompasses the fields of medicine and biomedical sciences, including pathology, physiology, immunology, pharmacology, respiratory medicine, cardiovascular medicine, nephrology, diabetes, as well as antioxidants and redox signaling. The yellow cluster focuses on pharmacology, toxicology, biochemistry, and biophysics. Key journals within this cluster include Biochemical and Biophysical Research Communications and Biochemical Journal. The purple cluster represents the field of neuroscience, with the Journal of Neurochemistry leading the cluster. These journals have made significant contributions to promoting Nrf2 research.

According to the analysis shown in Figure 5D, in terms of centrality, Journal of Biological Chemistry ranked first with a centrality value of 0.83, followed by Proceedings of The National Academy of Sciences of The United States of America with 0.72 and Free Radical Biology and Medicine with 0.28. These journals exhibited high centrality, indicating their significant impact in the field.

**FIGURE 6:**
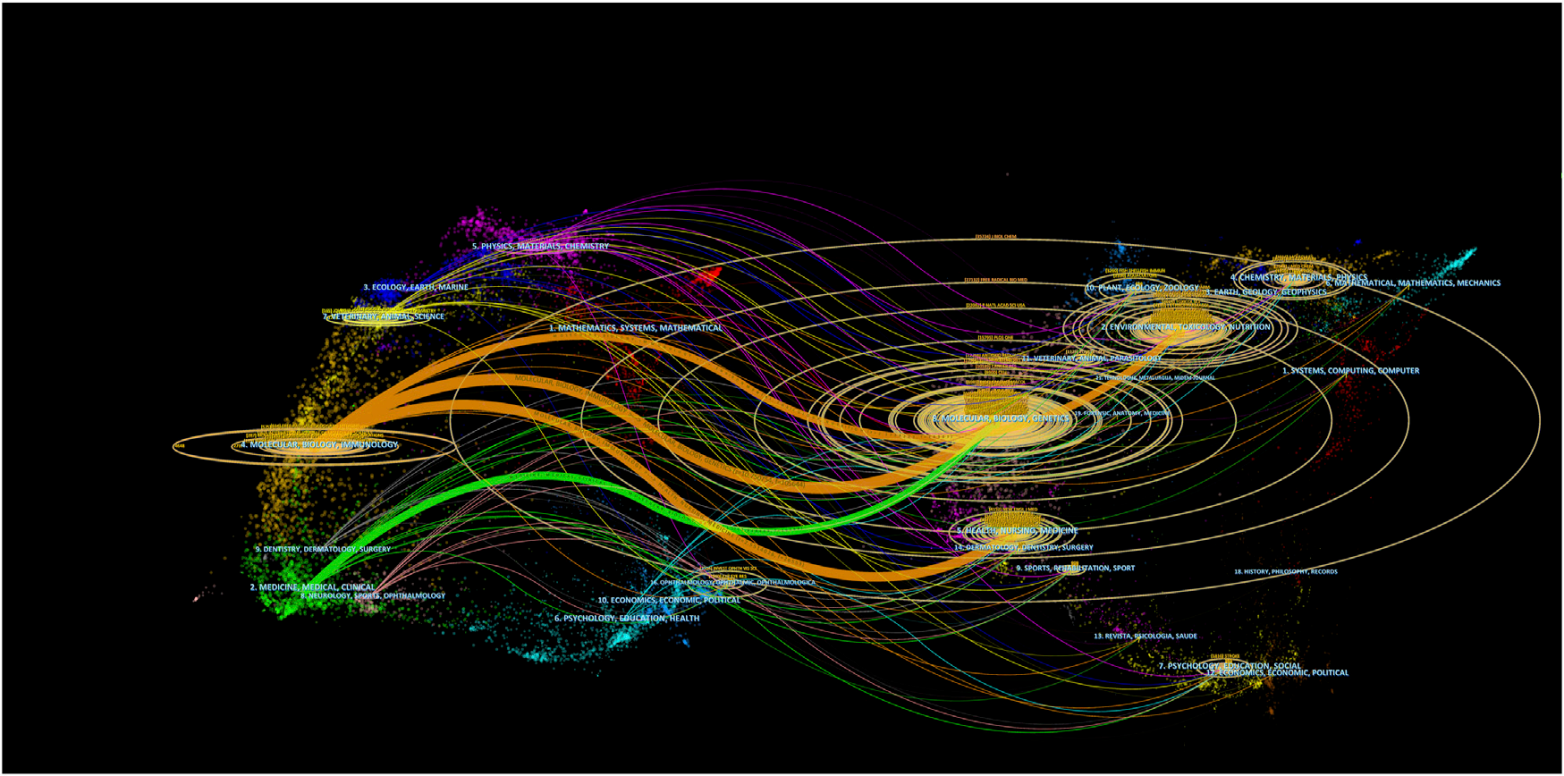
The dual-map visualization presents journals. Citing journals are positioned on the left, while cited journals are positioned on the right. The presence of citation relationships is represented by pathways colored in orange or green.

The dual-map visualization of journals provides valuable insights into topic distribution, the evolution of citation trends, and shifts in research emphasis among different scholarly publications. The labels on the left side of the dual map indicate the journals that are citing, while the labels on the right side indicate the journals being cited. The citation context is visually represented by a colored curve connecting the citing map to the cited map (Chen and Leydesdorff, 2013). Based on the dual-map overlay (Figure 6), two primary citation paths were observed. The publications primarily belonged to the fields of life sciences and healthcare, encompassing molecular, biological, and immunological areas, as well as medicine, medical, and clinical domains. Journals across various fields, including molecular biology, genetics, environmental science, toxicology, nutrition, health, nursing, medicine, chemistry, materials science, and physics, featured the most frequently cited articles.

### 3.5 Analysis of Co-authorship and core authors

The VOSviewer analysis also included the cooperation network of authors, revealing that 96,136 authors were involved in Nrf2-related studies.

**FIGURE 7:**
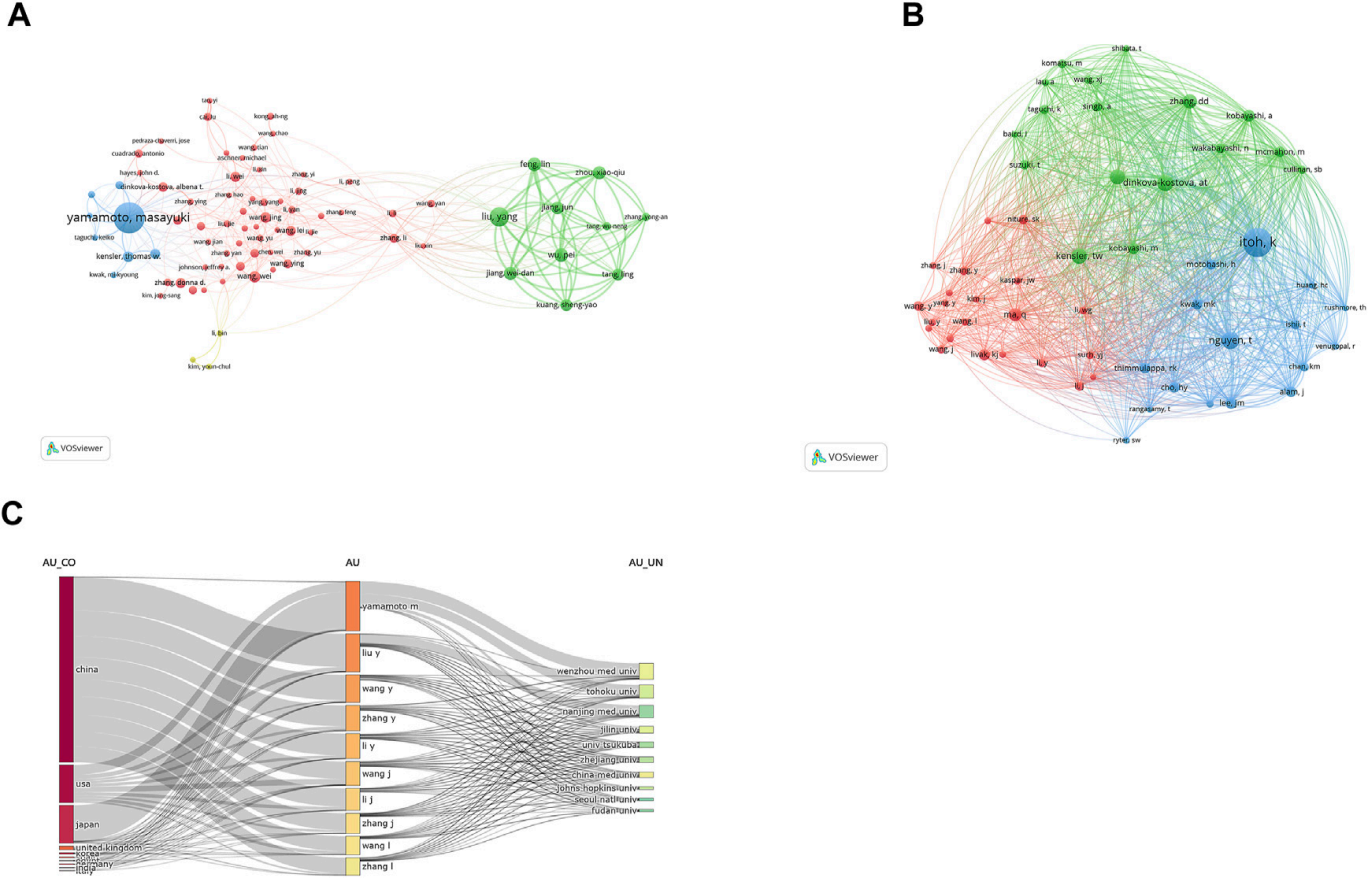
Authors active in the study of Nrf2. **(A)** The cooperation network of co-occurring authors visualized using VOSviewer. **(B)** Co-cited authors. **(C)** Three-field plot of the authors analysis on Nrf2. Notes: three-field plot of the authors analysis (middle field: authors; left field: countries; right field: institutions).

Table 5 presents the top ten core authors and their corresponding data in terms of publications and citations. A minimum of 37 articles published by an author was set as a criteria, and 78 authors met the criteria. Figure 7A illustrates the division of the co-authorship network into five distinct clusters, each identified by a unique color. Within the figure, individual circles symbolized authors, while the lines connecting the circles indicated the interconnections among them. Different colors showed the cooperation cluster between authors. Masayuki Yamamoto held the top position in terms of publications with 307 articles, followed by Liu Yang with 166 articles, Feng Lin with 113 articles, and Wu Pei with 107 articles. It is evident that the author centrality was relatively low, with only two authors having centrality values of 0.05 or higher.

**TABLE 5:** The top 10 authors by publication and citation count related to Nrf2 research.

Rank	Author	Count	Centrality	Co-cited author	Citation	Centrality
1	Yamamoto Masayuki	307	0.13	Itoh K.	3669	0.90
2	Liu Yang	166	0.00	Nguyen T.	2148	0.00
3	Feng Lin	113	0.05	Kensler T. W.	1981	0.02
4	Wu Pei	107	0.04	Dinkova-Kostova A. T.	1857	0.11
5	Jiang Weidan	100	0.00	Hayes J. D.	1728	0.39
6	Zhou Xiaoqiu	97	0.00	Ma Q.	1559	0.02
7	Jiang Jun	97	0.00	Zhang D. D.	1504	0.01
8	Tang Ling	89	0.02	Livak K. J.	1358	0.01
9	Kuang Shengyao	88	0.02	Kobayashi A.	1340	0.46
10	Wang Wei	81	0.02	Kobayashi M.	1280	0.01

When setting a minimum threshold of 675 citations per author, a total of 55 individuals were identified as co-cited authors, indicating that they were cited simultaneously in publications (Figure 7B). Itoh K. emerged as the top-ranked author with the highest number of citations, totaling 3669. Nguyen T. claimed the second position with 2148 citations, while Kensler T. W. secured the third position with 1981 citations. Notably, among these authors, Itoh K. displayed a remarkable centrality value of 0.90, followed by Kobayashi A. with 0.46, Hayes J. D. with 0.39, and Dinkova-Kostova A. T. with 0.11, indicating their significant influence and connectivity within the research network.

Figure 7C depicts the interconnected relationships among countries, authors and institutions within the field of Nrf2 research. In summary, a significant proportion of authors, 90% to be precise, have affiliations with institutions in China. Moreover, out of the 10 institutions represented, 6 of them are Chinese, indicating a strong presence and contribution from China in the field.

### 3.6 Highly-cited articles

**FIGURE 8:**
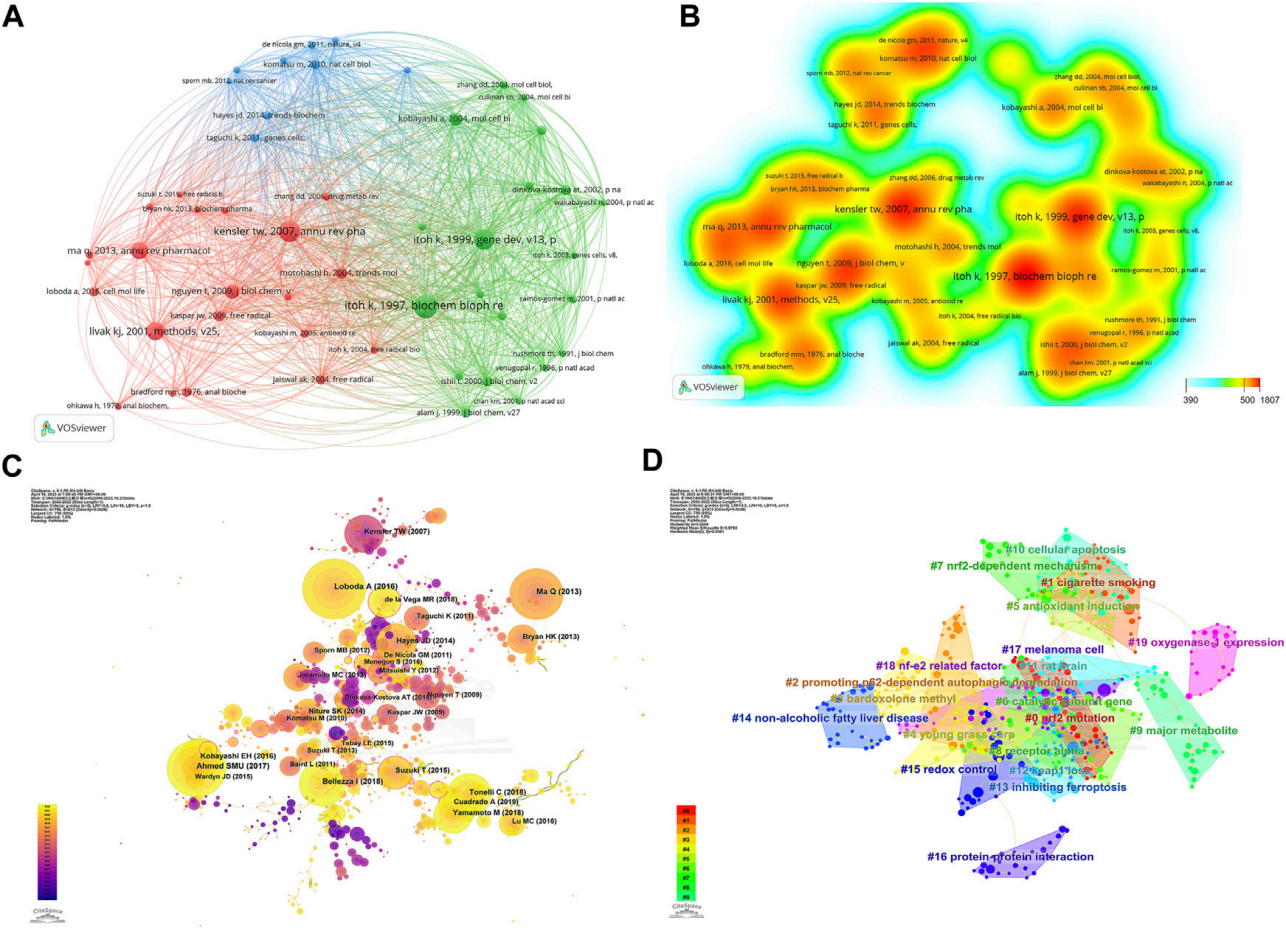
Co-cited references concerning Nrf2. **(A)** Reference co-citation network. The circles depicted in the visualization represent the co-cited literature, revealing the relationships and connections among the referenced articles. **(B)** Map illustrating the density of co-cited references. **(C)** Graphical representation of the network structure of cited references. **(D)** Visual depiction of the network structure of co-cited references related to Nrf2 research, organized into distinct clusters.

A co-citation network arises from examining the citation relationships among literature, while conceptual clusters gather related papers that share common citation patterns within this network. Co-citation networks investigate how literature references are cited by multiple papers, and conceptual clusters unite papers with similar citation patterns. Based on Figures 8A, B, the most cited article by K Itoh, titled “An Nrf2/small Maf heterodimer mediates the induction of phase II detoxifying enzyme genes through antioxidant response elements” revealed the critical role of the Nrf2/small Maf heterodimer in activating phase II detoxifying enzyme genes. This heterodimer combines with antioxidant response elements (AREs) in DNA and regulates the genes associated with detoxification and antioxidant defense mechanisms.

In Figure 8C, the network visualization map depicts the connections among cited references, where nodes symbolize the references and links represent co-citation associations. The size of the nodes correlates with the number of co-citations, while the color gradient (ranging from purple to yellow) signifies distinct years spanning from 2000 to 2022. CiteSpace categorized the relevant clusters into 20 groups (shown in Figure 8D), encompassing various topics such as Nrf2 mutation, cigarette smoking, p62-dependent autophagic degradation, bardoxolone methyl, young grass carp, antioxidant induction, catalytic subunit gene, Nrf2-dependent mechanisms, alpha receptor, major metabolite receptor, cellular apoptosis, rat brain, Keap1 loss, inhibiting ferroptosis, non-alcoholic fatty liver disease gene, redox control, protein-protein interaction, melanoma cell, nf-e2 related factor, and oxygenase-1 expression.

**FIGURE 9:**
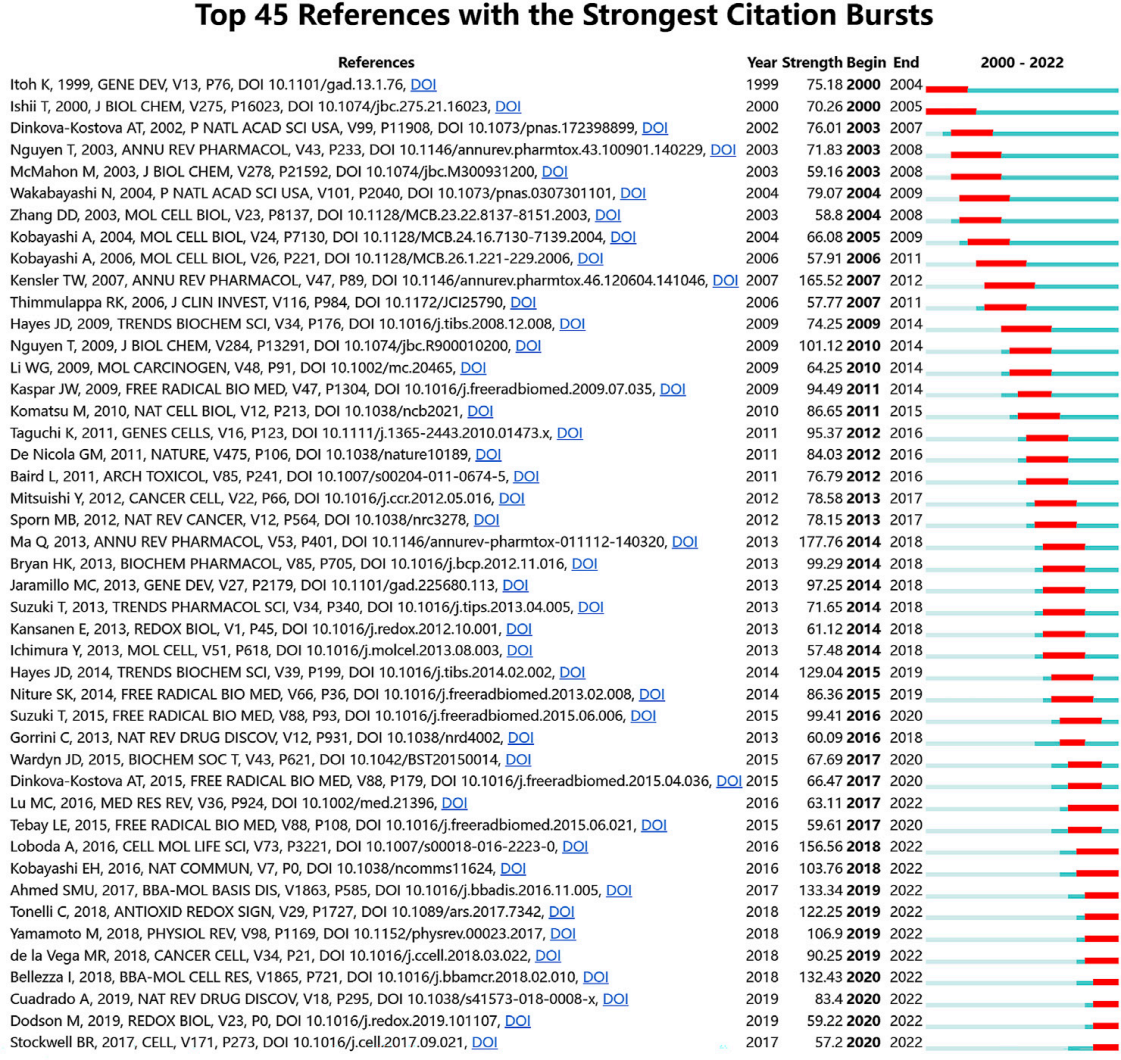
The 45 references that experienced the most significant surges in citations. The red bars indicate the durations of the bursts, while the strength denotes the degree of citation intensity for the literature.

A sudden and remarkable increase in the number of citations received by specific articles within a defined time period is referred to as a citation burst. It serves as a valuable tool for identifying emerging research topics and gaining insights into current trends within relevant academic domains (Miao et al., 2022). We identified the top 45 references that experienced a highest citation burst (Figure 9). The blue line depicts the time span, while the red line represents the surge duration, illustrating the duration of the citation surge within a specific time period. The first two citation burst began in 2000, entitled “Keap1 represses nuclear activation of antioxidant responsive elements by Nrf2 through binding to the amino-terminal Neh2 domain” (Itoh et al., 1999) and “Transcription factor Nrf2 coordinately regulates a group of oxidative stress-inducible genes in macrophages” (Ishii et al., 2000). The article “Role of nrf2 in oxidative stress and toxicity” (Ma, 2013) by Qiang Ma, published in the Annual Review of Pharmacology and Toxicology in 2013, had the highest citation burst with a strength value of 177.76. The burst occurred from 2014 to 2018. Another significant citation burst was observed for the article “Cell survival responses to environmental stresses via the Keap1-Nrf2-ARE pathway” (Kensler et al., 2007) by Thomas W Kensler, also published in the same journal, with a strength value of 165.52. According to the findings, 2014 exhibited the highest frequency of new citation bursts, totaling 6 occurrences. This was subsequently followed by the years 2017, 2019, and 2020, each with 4 new citation bursts. These observations suggest that the papers with significant bursts in 2014, as well as in the recent 5 years, have contributed to a surge in related research activities. There are ten citation bursts until 2022, these articles collectively investigate the role of the Nrf2-Keap1 signaling, Nrf2/HO-1 system in development, cellular defense against oxidative stress, as well as its crucial involvement in inflammation, redox balance, tumors, chronic diseases, and cell death processes such as lipid peroxidation and ferroptosis. This indicates that research focused on Nrf2 is still actively being pursued.

### 3.7 Keyword analysis

**FIGURE 10:**
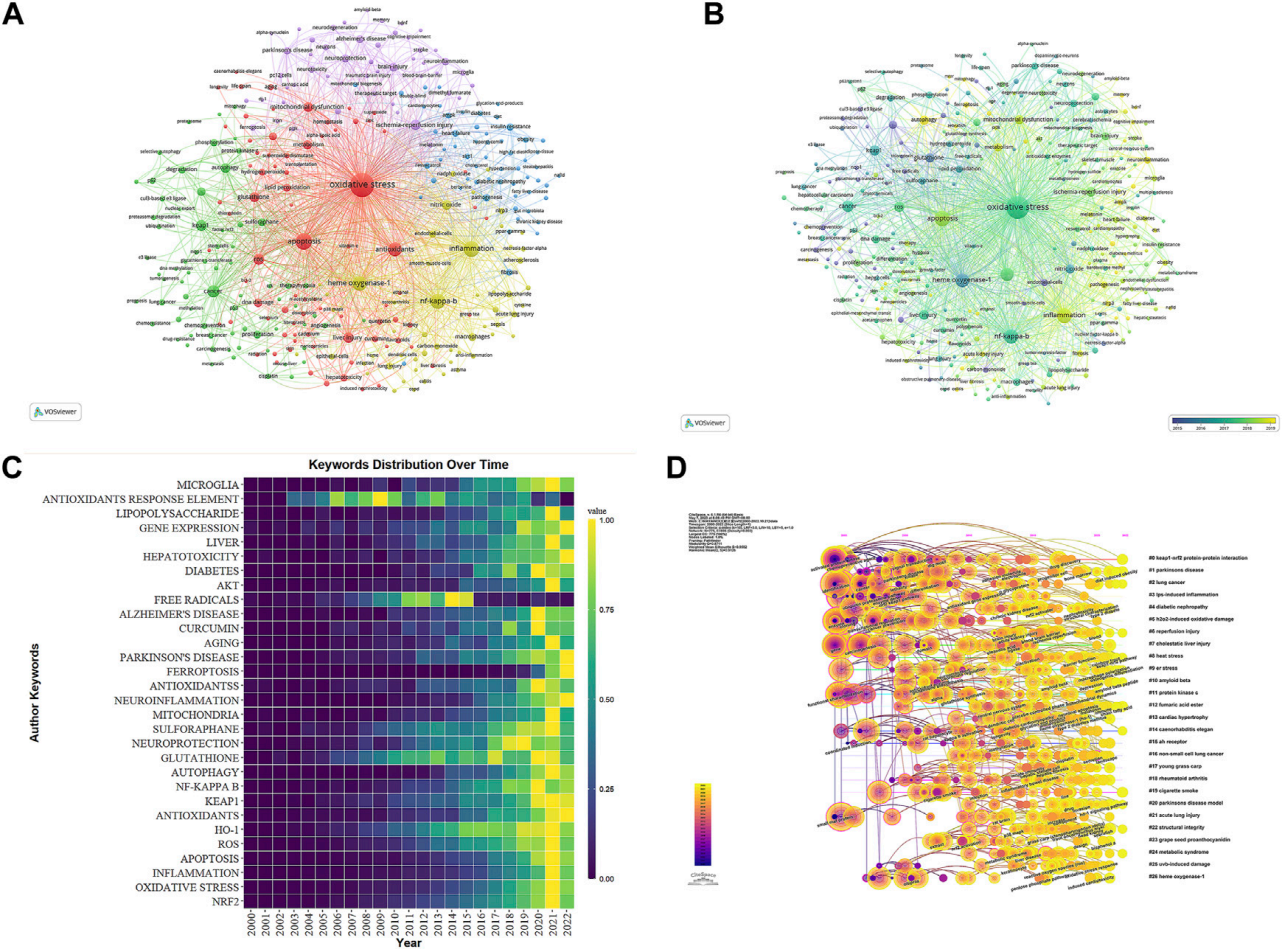
The representation of keyword mapping focusing on Nrf2. **(A)** The top 315 keywords were visually represented in a network visualization, with five clusters denoted by different colors (red for Cluster 1, green for Cluster 2, blue for Cluster 3, yellow for Cluster 4, and purple for Cluster 5) after removing irrelevant terms and merging synonyms. Node size reflects the frequency of occurrence. **(B)** Visualization of keywords based on density. **(C)** Occurrences of the top 30 keywords on a yearly basis. **(D)** In the timeline view, keyword clusters are displayed according to their co-occurrence in pathfinder pruning. Clusters are represented as horizontal lines, with their size inversely proportional to the assigned number, Cluster #0 represents the most dominant cluster, with the node size reflecting the frequency of co-occurrence. The connections among nodes signify the interactions of co-occurrence. Furthermore, the year of node occurrence signifies the initial co-citation.

Keywords offer a comprehensive analysis of the cutting-edge of Nrf2 research, serving as a summary of the article’s essential content. VOSviewer and CiteSpace were utilized to create visual charts. To streamline the keyword selection process, we eliminated irrelevant terms and combined synonymous terms. This ensured a more focused and comprehensive representation of the keywords. In total, 43,177 keywords were extracted from 22,040 documents. By analyzing the data, we identified a collection of 315 keywords based on their occurrence frequency, selecting those that appeared more than 62 times. The co-occurrence of keywords is visualized in a network diagram using VOSviewer. The connections between keywords indicate their relationships in terms of co-occurrence. The keywords are grouped into five clusters according to their research direction (Figure 10A). The red cluster, which is the most extensive, comprises 80 keywords related to oxidative stress, antioxidant defense mechanisms, apoptosis, mitochondrial dysfunction, regulation of metabolism and homeostasis, hepatotoxicity and nephrotoxicity, aging and longevity, DNA damage and repair, lipid peroxidation, and the effects of different substances (including drugs, environmental toxins, and natural compounds) on biological systems. The green cluster is dedicated to exploring cancer-related subjects, encompassing breast, colorectal, lung, and prostate cancers. It delves into autophagy, cell proliferation, tumor metastasis, angiogenesis, and protein degradation. Prominent components within this cluster include Keap-1, Nqo1, p53, and p62. Additionally, it discusses chemotherapy medications like cisplatin and natural compounds such as sulforaphane. The blue cluster focuses on metabolic disorders, cardiovascular and kidney diseases, lipid metabolism, insulin resistance, mitochondrial biogenesis, regulation of adipose tissue and skeletal muscle, diet and nutrition, as well as the molecular mechanisms and pathological processes associated with Ampk, SIRT1, and PPAR-gamma pathways. The yellow cluster pertains to inflammation and immune response, primarily in respiratory diseases. Key factors involved encompass heme oxygenase-1, NF-kappaB, NLRP3, p38 MAPK, PI3K/Akt, TNF-alpha, and Bach1. The purple cluster centers around neurological disorders such as ischemia-reperfusion injury, Alzheimer’s disease, and Parkinson’s disease. It also encompasses neurotrophic factors, iron ions, and iron-induced cell death. Notable factors within this cluster include BDNF, DJ-1, PI3K, and others. The overlay map of keywords, organized by publication year, demonstrates the dynamic shifts in research focus over time (Figure 10B). More recent keywords, such as ferroptosis (2021.01), pyroptosis (2020.97), and gut microbiota (2020.80), are highlighted on the map, while keywords like NLRP3 (2019.95), polysaccharides (2019.86), mitophagy (2019.84), NAFLD (2019.83), and osteoarthritis (2019.69) are relatively less recent. The color spectrum in Figure 10C, ranging from purple to yellow, reflects the varying popularity of top 30 keywords over a span of 20 years. Yellow hues indicate a higher frequency and greater significance, while purple shades suggest a diminishing level of importance.

In addition to the Citespace analysis, we identified and presented the most prominent 27 clusters of keywords associated with Nrf2 in Figure 10D. These clusters encompass diverse research domains and shed light on the main focuses within the academic community. During the period from 2000 to 2010, Nrf2 literature primarily concentrated on oxidative stress, antioxidant defense, tumor biology, inflammation and immune regulation, as well as neuroprotection and neurodegenerative diseases. Subsequently, Nrf2 research advanced its comprehension of signal pathways, regulatory mechanisms, and therapeutic drugs in these areas from 2011 to 2022. It progressively broadened its scope to encompass additional fields, such as chronic diseases (e.g., cardiovascular diseases and diabetes) and metabolic regulation (including energy, lipid, and glucose metabolism). Furthermore, there was an exploration of environmental toxicity and exposure to chemical substances in the research.

## 4 Discussion

### 4.1 Global trends in Nrf2

To our knowledge, this study pioneers a thorough and systematic bibliometric analysis exploring the basis and research frontiers of Nrf2, covering a wide range of aspects. In this research, VOSviewer, CiteSpace, and R software tools were employed to examine the research patterns and primary areas of emphasis concerning Nrf2 over the last two decades in the Web of Science Core Collection. In this study, we conducted an analysis of a comprehensive dataset consisting of 22,040 articles originating from 11,419 institutions across 117 countries. The involvement of 96,136 authors was recorded, and the articles were published in a total of 1,927 journals.

During the period from 2000 to 2021, there was a remarkable increase in the annual publication output and citation frequency of Nrf2 research. This upward trend became more pronounced after 2018, indicating a growing interest and significant impact of Nrf2 in the scientific community. Asia, Europe, and North America lead in Nrf2 research publications, with China making notable contributions to the field, with the United States and South Korea coming after. Remarkably, China accounted for the majority of the top 10 prolific institutions in Nrf2 research, including renowned institutions like China Medical University, Chinese Academy of Science, and Nanjing Medical University. And Johns Hopkins University stands out with the highest average citations and H-Index, followed closely by Tohoku University higher value. Except that, the United States had higher H-index and average citation values compared to China, indicating the exceptional research output. While China has made remarkable progress and holds a prominent position in Nrf2 research, there is still potential for enhancing collaboration across regions and strengthening academic impact. The current distribution of research in this field at the international level reveals an imbalance, with most collaboration centered around China and the United States. Japan, South Korea, Germany, and the United Kingdom have also been primary collaborators, while cooperation with other countries remains relatively limited.

Research progress in the field of Nrf2 has been documented in a range of journals across diverse disciplines, underscoring the widespread interest in understanding Nrf2’s role in disease development and treatment. Studies focusing on Nrf2 are predominantly published in journals that span multiple fields, including biomedical and life sciences, molecular science and cell biology, pharmacology and drug research, food science and nutrition, as well as multidisciplinary research in general science, including Plos One and Scientific Reports. There are a large number of high-quality papers in Nrf2 research. Based on the Journal Citation report (JCR), the number of Q1 journals accounts for half of the top 20 journals. And most of the publishers are in the United States, Central and Western Europe. Among the top 20 journals with the highest publication count, Redox Biology exhibited the highest impact factor (IF) of 10.787, establishing itself as a prominent journal since its inception in 2013. Free Radical Biology and Medicine, with an impact factor of 8.101, emerged as the most prolific journal in its field, alongside Oxidative Medicine and Cellular Longevity and Antioxidants. Free radical biology and medicine, initiated by the Society of Redox Biology and Medicine (SFRBM), covers a wide range of fields, including signal transduction, oxidative stress, aging, metabolic regulation, mitochondrial function and antioxidant enzymes. It is worth mentioning that the publication of Antioxidant has surpassed that of other journals since 2018, indicating the magazine’s great potential in the field of Nrf2 research. As one of the top 20 journals cited most frequently, the Journal of Biochemistry stands out because of its high H index and G index. It covers many research fields, such as biochemistry, molecular biology, cell biology, bioinformatics and enzymology.

Masayuki Yamamoto is an eminent author in the field of Nrf2 research. He stands out for his significant contributions and holds the highest publication output in Nrf2 studies. His team was the first to describe the structure and regulatory mechanism of Nrf2, unveiling its pivotal role in antioxidant defense and cellular stress response. One of his influential articles is a comprehensive review that explores the mechanisms and functionalities of the KEAP1-NRF2 system. It emphasizes the essential involvement of NRF2 in maintaining cellular redox balance and investigates its regulatory mechanisms in antioxidant defense, detoxification metabolism, and cellular stress response, offering insights into both normal and pathological conditions (Yamamoto et al., 2018). Itoh K. rose to prominence as the leading author with the most citations and centrality value, followed by Nguyen T. and Kensler T. W. The groundbreaking research publication of Itoh K. titled “Keap1 inhibits the nuclear activation of antioxidant response elements by Nrf2 through interaction with the amino-terminal Neh2 domain" (Itoh et al., 1999) which appeared in Genes and Development in 1999, garnered significant attention and had a profound impact within the academic community at that time. In this paper, Keap1 is considered to act as a critical sensor alongside Nrf2 in the context of oxidative stress, playing a pivotal role in the signaling pathway, that activates transcription via Nrf2’s nuclear shuttling mechanism, which lays the foundation for further in-depth investigations into the function of Nrf2. Kensler T. W’s article titled “Cell survival responses to environmental stresses via the Keap1-Nrf2-ARE pathway" (Kensler et al., 2007) in the Annual Review of Pharmacology and Toxicology is one of the top 45 references with the strongest citation bursts. The article highlights the critical role of the Keap1-Nrf2-ARE signaling pathway in allowing cells to adapt and safeguard against acute and chronic cellular damage caused by environmental stresses.

### 4.2 Fundamental understanding, research trends, and promising areas in Nrf2 studies

This study provides a thorough of diverse research findings and areas of interest observed over the last two decades. Additionally, it presents an overview of the advancements and trends within Nrf2 during this period. As diverse perspectives show in publication, keyword clustering, and highly-cited articles, we have successfully identified the principal research areas and focal points associated with Nrf2. These mainly encompass research into oxidative stress, cancer, glycolipid metabolic disorder, inflammation, and neurological disorder. The findings of these studies indicate that targeting Nrf2 activators as a therapeutic approach holds considerable potential.

#### 4.2.1 Involvement of oxidative stress

“Oxidative stress” is a term coined by Sies and Cadenas (1985) that illustrates the imbalance arising from the production of oxidants and the capacity of antioxidant defense mechanisms to counteract them. Oxidative stress refers to the inability of cells to effectively counteract reactive oxygen species (ROS), leading to cellular damage and dysfunction when there is an excess of ROS or inadequate antioxidant defense (Kobayashi et al., 2009; Forman and Zhang, 2021). This imbalance has the potential to cause harm to biological systems. Nrf2 is closely associated with oxidative stress and pivotal in cellular antioxidant responses.

Activating Nrf2 increases the expression of downstream antioxidant enzymes, facilitates the removal of toxic metabolites, and enhances the cellular defense against oxidative stress (Cescon et al., 2015; Matzinger et al., 2018; Liu et al., 2019). Additionally, Nrf2 regulates other redox-related biological processes, such as maintaining optimal levels of glutathione. Impairment of Nrf2 signalling or reduced Nrf2 activity has been observed in various pathological conditions associated with oxidative stress, including neurodegenerative diseases (Osama et al., 2020), cardiovascular diseases (Silva-Palacios et al., 2016), metabolic disorders (Zhang et al., 2015), and cancer (Schmidlin et al., 2021).

#### 4.2.2 Nrf2 and cancer

An increasing body of research indicates that Nrf2 possesses capabilities beyond its role in redox regulation. Over the past two decades since its discovery, Nrf2 has emerged as an indispensable player in cancer prevention and treatment. Nrf2 can maintain the redox dynamic balance of cells and exert its anti-inflammatory and anticancer activity, thus supporting cell survival under physiological conditions. Therefore, the activation of Nrf2 is crucial in cancer chemoprevention. Numerous *in vitro* and *in vivo* studies have shown that activation of Nrf2 exerts protective effects against the occurrence, initiation, and progression of cancer. The loss of Nrf2 in mice leads to an increased incidence of tumors (Kensler et al., 2007; Tao et al., 2018), and the liver-specific activation of Nrf2 controls fibrosis and carcinogenesis in non-alcoholic fatty liver disease (Mohs et al., 2021). And Compared to Nrf2 wild-type mice exposed to urethane, Nrf2 knockout mice developed a greater number of lung tumors (Satoh et al., 2010). Moreover, an increasing number of studies indicate the relationship between Nrf2 and aging. The expression of Nrf2 is negatively correlated with age, providing a plausible explanation for the higher susceptibility to cancer among the elderly population (Suh et al., 2004; Suzuki et al., 2008). Nrf2 activators have naturally emerged as a prominent area of research. Extensive studies have focused on various types of Nrf2 activators, including natural compounds derived from plants (such as sulforaphane (Lu et al., 2021), curcumin, resveratrol (Farkhondeh et al., 2020), EGCG, quercetin, and hesperidin), synthetic compounds (like bardoxolone methyl, dimethyl fumarate, and synthetic triterpenoids), electrophilic compounds (such as tert-butylhydroquinone and cinnamic aldehyde), metal ions (like zinc and selenium), and specific drugs (such as oltipraz and dimethyl fumarate). Since the first Nrf2 inducer DMF was approved for the treatment of multiple sclerosis, the discovery of KEAP1-NRF2 PPI inhibitors has become a very interesting alternative to the development of Nrf2 inducers with potentially safer clinical applications (Crisman et al., 2023).

On the contrary, an excessive activation of Nrf2 can grant cancer cells numerous benefits. Such as, promoting the uncontrolled growth of cancer cells (Rojo et al., 2014; Li et al., 2019). For instance, it enables cancer cells to become more resistant, avoiding apoptosis and senescence, while enhancing resistance to chemotherapy and radiation therapy (Sporn and Liby, 2012; Jaramillo and Zhang, 2013; Kansanen et al., 2013; Menegon et al., 2016). Various factors, such as chronic toxic exposure, protein-protein interaction, epigenetic modification (Bi et al., 2021), transcription/translation regulators, post-translational modification and mutation, have been shown to activate Nrf2 chronically in cancer, thus promoting its occurrence, development and metastasis. In such situations, inhibition of Nrf2 translation is necessary. Blocking Nrf2 signal pathway is expected to be a therapeutic method for cancers with elevated Nrf2 levels. Consequently, a diverse range of Nrf2 inhibitors has emerged, including Keap1 activators (e.g., brusatol (Ren et al., 2011), trigonelline), PI3K/Akt pathway inhibitors (e.g., LY294002 (Wu et al., 2020), Wortmannin), GSK-3β inhibitors (e.g., LiCl(Mathur et al., 2018)), mTOR inhibitors (e.g., rapamycin, everolimus), proteasome inhibitors (e.g., bortezomib, MG-132), and ROS scavengers (e.g., N-acetylcysteine, vitamin C (Wu et al., 2022)). However, caution should be exercised when using Nrf2 inhibitors due to variations in their applicability depending on specific conditions or diseases. Consulting with a healthcare professional or conducting further research is advised before considering the use of Nrf2 inhibitors.

Reasonable activation of Nrf2 in healthy tissues is still a promising chemoprevention strategy. However, excessive expression of Nrf2 can drive tumor invasiveness and induce therapy resistance. Hence, the task to be solved urgently lies in comprehending the intricate regulation and dual functionality of Nrf2 at various phases of cancer. This further emphasizes the need to consider these intricate characteristics when developing Nrf2-targeted drugs for combating cancer. This presents new opportunities and challenges for targeted Nrf2-based therapies in cancer.

#### 4.2.3 Nrf2 and glycolipid metabolic disorder

Glycolipid metabolism involves the synthesis, breakdown, and regulation of glycolipids, which are carbohydrates linked to lipids and play critical roles in cell membranes and various cellular functions. Abnormalities in glycolipid metabolism are associated with diseases such as hypertension, obesity, fatty liver, heart disease, diabetes and its complications, highlighting the importance of understanding and studying this metabolic pathway for potential therapeutic interventions.

Nrf2 activation has been found to enhance insulin sensitivity and promote glucose uptake by increasing the expression of key glucose transporters, such as GLUT4, in adipose tissue and skeletal muscle. This can lead to improved glucose utilization and reduced blood glucose levels. Obesity-induced lipotoxicity leads to insulin resistance. Benzyl isothiocyanate enhances Nrf2-dependent antioxidant defense, mediates skeletal muscle IRS-1/AKT/TBC1D1 signalling, and improves GLUT4 expression, thereby ameliorating high-fat diet-induced hyperglycemia (Chuang et al., 2020). Furthermore, NRF2 activation has been linked to the regulation of adipocyte differentiation (Zhang et al., 2015; Han et al., 2018) and adipokine secretion, which play crucial roles in metabolic regulation. Nrf2 signal transduction also directly upregulates key pro-adipogenic factors, such as CCAAT/enhancer binding protein-α C/EBP-α) and peroxisome proliferator-activated receptor γ (PPAR γ) (Pi et al., 2010; Kim et al., 2018). Some studies have found that 0.1% OI can regulate the expression of PPAR γ through Nrf2 and enhance glycolysis and lipogenesis in the liver (Fu et al., 2022). Except that, it can also influence the secretion of adipokines, such as adiponectin and leptin, which are involved in insulin sensitivity and energy homeostasis (Yagishita et al., 2017). Resveratrol inhibits pulmonary artery smooth muscle cell proliferation and right ventricular remodeling in pulmonary hypertension by regulating Nrf2, SIRT1 pathway and HIF-1α expression (Mirhadi et al., 2021). By upregulating the HO-1/Nrf2 pathway, the application of low molecular weight blue mussel hydrolysate impedes the differentiation of mouse mesenchymal stem cells into fat cells, thus improving the condition of obesity. (Oh et al., 2020). Sulforaphane protects against ferroptosis and its related diabetic cardiomyopathy by activating Nrf2 through AMPK signaling pathway (Wang X. et al., 2022).

In general, the activation of Nrf2 seems to have beneficial effects on glucose and lipid metabolism, making it a promising candidate for the relief and management of metabolic disorders. Further research demands deeper exploration the precise mechanism of Nrf2-mediated regulation of lipid metabolism and its therapeutic significance.

#### 4.2.4 Inflammation

Oxidative stress and inflammation are closely related, and they influence each other. Reactive oxygen species, activated white blood cells, macrophages and resident cells can cause oxidative stress (Himmelfarb et al., 2002; Cachofeiro et al., 2008). By activating NF-κB, a redox-sensitive transcription factor that regulates the expression of pro-inflammatory cytokines and chemokines, oxidative stress promotes recruitment and activation of leukocytes and resident cells on the one hand, and forms lipid oxides and advanced protein oxidation and glycation end products on the one hand, leading to inflammation (Cachofeiro et al., 2008).

Activation of Nrf2 can inhibit the inflammatory response. It regulates the production of inflammatory mediators, modulates the activity of immune cells, and improves the intracellular oxidative stress status, thereby attenuating the inflammatory process. Nrf2 activation can also suppress inflammatory signalling pathways, such as the NF-κB pathway, resulting in reduced production of inflammatory mediators and cellular damage (Wang Z. et al., 2023; Li et al., 2023) and save mitochondrial function by regulating NLRP3 inflammatory bodies (Rajan et al., 2023). In COPD, the inherent loss of metabolic plasticity leads to metabolic failure and decreased redox capacity, which can be rescued by activating the Nrf2 pathway (Ryan et al., 2023). Disruption of Nrf2 in mice enhances ovalbumin sensitization and exacerbates asthma responses (Rangasamy et al., 2005). Hydnocarpin D mitigates oxidative stress and inflammation in the context of LPS-induced acute lung injury by regulating the MAPK/NF-κB and Keap1/Nrf2/HO-1 pathway (Hong et al., 2022). By activating Nrf2, Galectin-1 attenuates LPS-induced lung injury (Huang et al., 2020).

Therefore, activation of the Nrf2 pathway can suppress inflammation and provide cellular protection and repair mechanisms, showing potential in managing and averting diseases associated with inflammation.

#### 4.2.5 Nrf2 and neurological disorder

In recent years, people are drawn to the role of Nrf2 in nervous system diseases. Studies have shown that Nrf2 activation plays a neuroprotective role in neurological diseases such as Parkinson’s disease (Bento-Pereira and Dinkova-Kostova, 2021) and multiple sclerosis (Montes Diaz et al., 2018). Nrf2 can regulate glucose uptake and metabolism of neurons (Esteras et al., 2023), autophagy (Li et al., 2021), ferroptosis (Song et al., 2023), inflammation (Wang J. et al., 2023), mitochondrial function (Jamwal et al., 2021), and promote energy metabolism (Esteras et al., 2023), which helps to maintain the health and survival of neurons. For example, CMPB can improve learning and memory impairment caused by fatigue by regulating PI3K/NRF2/HO-1 signal pathway, scavenging free radicals and fatigue metabolites, increasing the content of glycogen and regulating the content of central neurotransmitters in the body (Bai et al., 2023). Pharmacological or genetic activation of Nrf2 has shown promising therapeutic potential in preclinical models of neurological disorders (Li et al., 2021). At present, there are many phytochemicals that target Nrf2 signaling pathways to combat neurodegenerative diseases (Tian et al., 2022). Impaired Nrf2 signal can lead to a variety of nervous system diseases. In the early brain injury induced by intracerebral hemorrhage (ICH), the injury volume of Nrf2 (−/−) mice was significantly larger than that of WT control group (Wang et al., 2007). And impaired Nrf2 signal transduction may lead to oxidative stress in Friedreich ataxia (Paupe et al., 2009; Cook et al., 2011; Johnson and Johnson, 2015). Except that, genetic and epigenetic alterations, environmental toxins (Wei et al., 2022), and aging (L'Episcopo et al., 2013) can disrupt Nrf2 activity, leading to increased vulnerability to programmed cell death in nervous system diseases, such as ferroptosis (Wang T. et al., 2022).

Understanding the intricate involvement of Nrf2 in neurological disorders provides valuable insights into the development of novel therapeutic strategies that target Nrf2 activation and enhance endogenous neuroprotective mechanisms. However, further research is needed to elucidate the specific molecular pathways and mechanisms by which Nrf2 exerts its neuroprotective effects and to translate these findings into effective clinical interventions.

## 5 Limitations

It is important to take into consideration recent primary research and non-English studies in future research endeavors, as there is a possibility of language bias due to the English-only publications that were analyzed in this study. Additionally, the study solely utilized the WOS core database, and did not include other databases like PubMed, Google Scholar, and Scope. Furthermore, altering the bibliometric data at different time points may result in varying conclusions. Despite the relatively comprehensive and unbiased information on bibliometric and visualized analysis pertaining to Nrf2 research provided by this study, certain limitations still exist.

## 6 Conclusion

**FIGURE 11:**
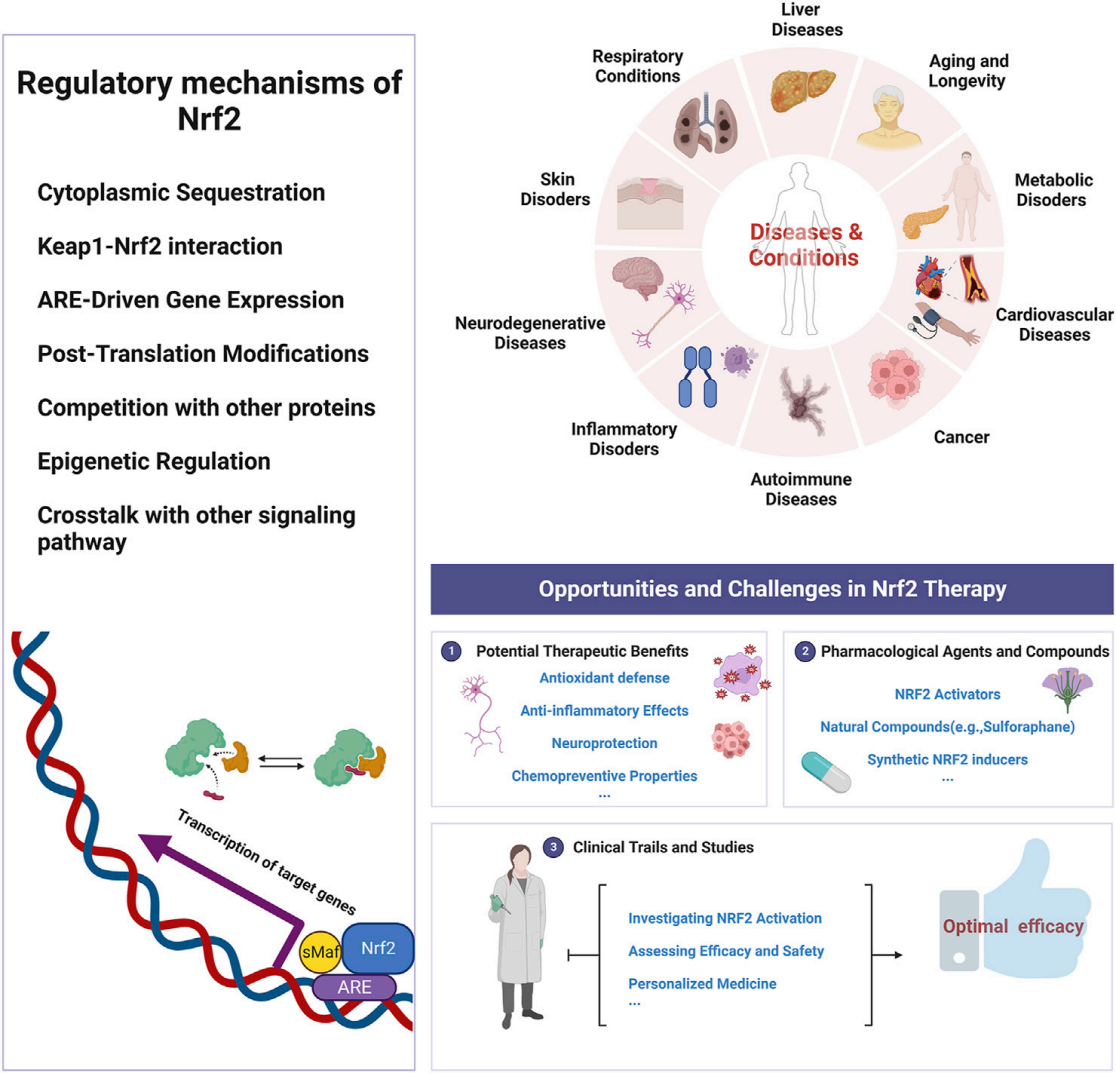
The summative insight map of Nrf2.

Unlike earlier bibliometric articles concerning Nrf2, which solely concentrate on the impact of natural compounds modifying its function and its role in inflammation and cancer studies. This research offers the relatively comprehensive analysis of Nrf2-related studies, covering productive authors, countries, regions, institutions, and journals. It also examines the current state, emerging trends, and areas of focus within the field. China and the United States have emerged as leaders in Nrf2 research. Recently, research on Nrf2 has focused on understanding its role in various physiological and disease processes. Future directions in Nrf2 research will focus on advancing our understanding of its novel mechanisms, specifically related to NF-κB, Ho1, and Keap1. The new trend of Nrf2 in the treatment of diseases may involve the following aspects: 1) Mechanistic research: conducting in-depth studies into the mechanistic role of Nrf2 in various diseases, providing a deeper understanding for treatment strategies. 2) Drug Development: designing and developing novel Nrf2 modulators to improve effectiveness, specificity and reliability in treatment. 3) Targeted therapeutic strategies: using Nrf2 modulators for disease treatment. 4) Combination therapy: employing Nrf2 modulators in combination with other drugs or treatments to enhance efficacy or minimize adverse reactions. 5) Personalized treatment: incorporating Nrf2 modulators into personalized treatment plans based on patients’ genetic and phenotypic characteristics. 6) Clinical trials and application: assessing the efficacy of Nrf2 modulators through clinical trials and evaluating their real-world effectiveness in clinical practice. In summary, our study provides a broad overview of the research patterns in Nrf2, offering valuable guidance and aiding researchers in identifying future research directions in this field. The summative insight map is depicted in Figure 11.

## Data Availability

The original contributions presented in the study are included in the article/Supplementary material, further inquiries can be directed to the corresponding authors.
